# Effects of chronic workplace harassment on mental health and alcohol misuse: a long-term follow-up

**DOI:** 10.1186/s12889-023-16219-0

**Published:** 2023-07-26

**Authors:** Kathleen M. Rospenda, Judith A. Richman, Meredith McGinley, Kristin L. Moilanen, Tracy Lin, Timothy P. Johnson, Lea Cloninger, Candice A. Shannon, Thomas Hopkins

**Affiliations:** 1 Department of Psychiatry, University of Illinois at Chicago, Chicago, IL USA; 2 Department of Psychology, Professional Counseling, and Neuroscience, University of WI – Parkside, Kenosha, WI USA; 3 NORC at the University of Chicago, Chicago, IL USA; 4 Department of Sociology, University of Maryland, College Park, MD USA

**Keywords:** Occupational stress, Sexual harassment, Generalized workplace harassment, Workplace bullying, Mental health, Alcohol misuse

## Abstract

**Background:**

Research on the impacts of exposure to workplace harassment (WH) is largely cross-sectional, and existing prospective studies generally are between two and five years of follow-up, with the longest US study being 10 years. However, the effects of exposure to WH may persist longer, particularly if exposure has been chronic. This study fills this gap by examining effects of prior exposure to chronic sexual and generalized WH on psychological distress and alcohol misuse over an approximately 25 year study period.

**Methods:**

Individuals originally recruited from a university-employed sample in the United States were surveyed at 8 time points from 1996–2007 and again in 2020–2021. A series of hybrid path analyses were tested on a sample of 2352 individuals, regressing recent outcomes on latent classes of harassment derived from earlier survey waves, controlling baseline outcomes and demographics. Model fit was assessed using a variety of fit statistics, and standardized regression coefficients were used to assess significance of individual pathways.

**Results:**

Prior exposure to chronic sexual harassment had significant direct associations with psychological distress, alcohol misuse, and recent stressors at follow-up. Prior exposure to chronic generalized harassment had significant direct associations with lower income and alcohol misuse. Both forms of WH were significantly indirectly associated with psychological distress through recent stressors at follow-up.

**Conclusions:**

Exposure to chronic WH is associated with long-term effects on psychological distress and alcohol misuse in a sample representing a wide variety of job types and racial/ethnic identities. Enforcement of anti-sexual harassment law and policies and enactment of policies and laws to prevent generalized harassment/workplace bullying are imperative for the protection of worker health.

**Supplementary Information:**

The online version contains supplementary material available at 10.1186/s12889-023-16219-0.

## Background

In a study of the United States (U.S.) civilian labor force, Goh et al. [1] estimated that roughly 120,000 deaths and 5–8% of U.S. yearly healthcare costs can be attributed to psychosocial workplace exposures (e.g., job demands, organizational injustice), adding to the large body of research on shorter term health-related effects of psychosocial workplace stressors. However, absent from their model is workplace harassment (WH). WH includes *sexual harassment* (SH)—unwelcome sexual conduct that affects an individual’s job status or creates a hostile/offensive work environment [2] and *generalized harassment*(GH)—interpersonal mistreatment not based on gender or other legally protected characteristics (e.g., race/ethnicity, age). GH is very similar to the concept of workplace bullying. Both encompass experiences such as verbal aggression, disrespect, exclusionary behavior, and threats, but GH does not require harmful intent of the perpetrator or a specified duration of experiences [3]. Because workplace bullying is a closely overlapping construct with GH, it informs our review. Research suggests WH is a traumatic occupational stressor that tends to be chronic in nature [4–6], and may be a stronger predictor of health-related outcomes (e.g., alcohol use, depression, and anxiety) compared to other more commonly-studied work stressors (e.g., job demands, control, role conflict) [4, 7, 8]. Approximately 58% of women and up to 40% of men report SH experiences at some time in their work careers [9, 10], and about 60% of both women and men report GH or bullying experiences [7, 11, 12].

Research on the associations between mental health and WH is largely cross-sectional, and most of this research has been done outside of the U.S. This substantial body of research shows that both SH and GH are associated with decrements to mental health including increased depression, anxiety, and suicidal ideation [8, 13, 14]. Recent cross-sectional research continues to show associations between SH and greater symptoms of depression, anxiety, suicidality, or poor mental well-being in samples as varied as women soldiers in Belgium [15], men and women medical school faculty in the Midwestern US [16], men and women medical students in France [17] and Switzerland [18], and international humanitarian field workers [19]. Research on GH, or closely related constructs such as workplace bullying, is not as common as SH research. This is likely because GH is not legally prohibited in all countries and because of a resurgence in attention to SH following the #MeToo movement. However, as for SH, recent cross-sectional research continues to support an association between GH exposure and poor mental well-being in a variety of samples such as US public health professionals [20], US and Canadian flight attendants [21], US women firefighters [22], and National Health Service workers [23]. Although not cross-sectional, a short-term daily diary study in an occupationally diverse sample of full-time employees in Spain showed associations between an increasing trajectory of workplace bullying experiences and increased depression and anxiety over a four week period [24].

While alcohol use and misuse are less commonly studied by WH researchers, cross-sectional research supports an association between SH and GH and higher levels of alcohol use and misuse [7, 12, 25]. Recent research on WH and alcohol use has mainly focused on SH in military samples. In one study, there was no association between SH and alcohol misuse, possibly because of the methodological limitation of a single item to assess SH ever during one’s military career [26]. In another study that combined experience of multiple traumas, such as SH with combat trauma, those who experienced both combat trauma and SH in the military reported the highest past month alcohol use and highest scores on a measure of alcohol misuse compared to groups with other single or combined trauma exposures (e.g., combat trauma alone, childhood trauma plus combat trauma) [27]. Similarly, men in the US Army Reserve or National Guard who experienced sexual harassment or assault (only *n*= 4 respondents reported sexual assault) in their most recent deployment exhibited significantly increased risk for heavy drinking and alcohol problems post-deployment [28]. In a non-military sample, US women firefighters who reported a higher frequency of exposure on a combined measure of work-related discrimination and harassment exhibited significantly higher odds of alcohol misuse [22].

The limited longitudinal research that has been conducted supports lingering detrimental effects of WH on mental health over various time frames, focusing mainly on depression. In follow-up studies, researchers have demonstrated associations between SH and elevated depressive symptoms two years later in a large study of men and women Danish workers [29] and elevated distress two years following SH for women in a representative sample of Norwegian workers [30]. Considering longer time frames, data from the U.S. Youth Development Study found that experiences of SH early in one’s career were associated with increased depressive symptoms up to 10 years later for both women and men [31]. Additionally, a national prospective study of Swedish workers that used data from national health registries found significant associations between exposure to SH and suicide attempts and completed suicides over an average follow-up period of 13 years for both women and men [32].

Similarly, a recent systematic review of longitudinal studies supports workplace bullying’s persistent negative effects on mental health. However only six of 54 studies reviewed examined mental health symptoms at follow-up periods of greater than two years, with the longest follow-up period being five years [33]. These six studies consistently show that exposure to workplace bullying is associated with increased symptoms of depression, anxiety, suicidal ideation, and diagnosis with a common mental disorder three to five years following exposure [34–39]. While compelling, these publications do not specifically address GH. Also, three of these publications derive from the same study, and none of these studies were conducted with U.S. workers or examine longer term effects.

A single U.S. prospective study has examined longer term impacts of both SH and GH on alcohol use and misuse in a sample of workers originally drawn from an urban university in the Midwest. This study found that experiences of chronic harassment predicted increased alcohol misuse over a 10 year period, controlling for earlier drinking [5]. Here, we present additional follow-up data from this sample, extending existing occupational health research on WH by examining the psychological and behavioral health effects of exposure to harassment over an approximately 25 year time span. Given the high prevalence of exposure to WH, it’s important to understand how exposure to WH may impact the longer-term health of workers, especially into later adulthood. We hypothesize that prior exposure to chronic harassment will be associated with greater symptoms of psychological distress and greater alcohol use/misuse over time. Demonstrating continuing deleterious effects of WH over a major period of workers’ lives would have profound implications for occupational safety and health policy and prevention efforts, and population health. Our study extends existing occupational health research by examining the effects of prior exposure to WH on recent symptoms of psychological distress and alcohol misuse over an approximately 25-year study period in a U.S. based sample.

## Methods

### Study sample

Respondents were originally drawn from a sample of 4832 university employees, stratified by gender (2416 men; 2416 women) and occupational group (faculty; graduate student workers/trainees; clerical/administrative workers; service/maintenance workers) based on university payroll classifications, to help ensure our final sample represented a variety of worker types. Workers were surveyed at nine different time points, or waves, throughout the study (Wave 1 – Wave 9, or W1-W9). Workers were first surveyed in 1996–1997 (W1; *N* = 2492, 52% response rate at W1), resurveyed at 7 additional time points through 2007 (W2-W8), and again by web or mail between July 2020 – February 2021 (W9), approximately 25 years following W1. Those previously requesting removal from the study (*n* = 70) and known deceased (*n* = 35) were excluded from follow-up, for a possible W9 follow-up sample of *n* = 2387. At W9, an additional 37 past participants were found to be deceased, 3 were incapacitated (e.g., very ill) and unable to participate, 30 refused, and 374 were not locatable. Valid responses were received from *n* = 921 individuals at W9 (4 were disqualified due to W1-W9 demographics mismatch; 39.2% reinterview rate excluding known deceased or 38.6% including known deceased). The study was approved by the University of Illinois at Chicago IRB (protocol 2019–0374).

Demographic characteristics of the W1 sample were as follows: 53.7% women; average age of 40 years; 51.1% white, 21.1% Black, 7.6% Hispanic, 16.5% Asian, 2.3% other or more than one race/ethnicity. W1 occupational groups were 30.7% faculty; 22.4% clerical/administrative; 35.1% graduate student workers/trainees (RA/GA/TA/Medical Resident); 11.8% service/maintenance. Attrition analyses indicated those who were missing data at one or more waves after W1 were disproportionately members of the chronic SH class, Black, Hispanic, Asian, student or service/maintenance workers at W1 (Cramer’s V range = 0.05—0.08), were younger and had higher depression scores at W1 compared to those who were present for all study waves through W9 (both η^2^ = 0.003).

### Measures

*Sexual Harassment at Work* was measured at W1-W7 and at W9 with a modified version of the Sexual Experiences Questionnaire(SEQ) [40], with questions modified to be applicable to both women and men. The 19-item SEQ measures past 12-month experiences of gender harassment (e.g., told suggestive stories or offensive jokes), unwanted sexual attention (e.g., made unwanted attempts to stroke or fondle you), sexual coercion (e.g., implied faster promotions of better treatment if you were sexually cooperative), and a single item to measure sexual assault attempts (made unwanted attempts to have sex with you that resulted in you pleading, crying, or physically struggling). Respondents indicated the frequency of experiences as occurring never = 0, once = 1, or more than once = 2. Coefficient alpha reliability for the SEQ was 0.80 or higher at each wave.

*Generalized Harassment at Work* was measured at W1-W7 and at W9 with the 29-item Generalized Workplace Harassment Questionnaire(GWHQ) [3]. The GWHQ includes items from four conceptual areas of GH: covert hostility (e.g., being excluded from important meetings or events), verbal hostility (e.g., being yelled at, talked down to), manipulation (attempts at controlling the target’s behavior, e.g., through threats or bribes), and physical aggression (e.g., pushed, hit, kicked). Respondents indicated the past 12 month frequency of experiences as occurring never = 0, once = 1, or more than once = 2. Coefficient alpha reliability for the GWHQ was 0.90 or higher at each wave.

Due to study funding gaps, respondents were asked at W3 to recall SH and GH experiences during each of the three prior 12 month periods, and at W6 respondents were asked to report on the two prior 12 month periods. Thus, we had ten measurement points for SH and GH covering the 10-year W1-W7 period. Previously, we used growth mixture modeling with full-information maximum likelihood estimation for missing data to identify individuals with different developmental growth trajectories for GH and SH over time. For both SH and GH, two latent classes of harassment were extracted: *chronic* (a pattern of elevated mean levels of harassment across the 10 measurement points collected from W1-W7) and *infrequent* (a pattern of low mean levels of harassment across the 10 measurement points collected from W1-W7) [5]. The latent classes for SH and GH were used as predictors in our models (0 = infrequent/no harassment; 1 = chronic harassment).

A *Stress Index* to assess a count of various life stressors was computed by summing measures of stressors experienced in the 12 months prior to the W9 survey (1 = yes, 0 = no for all items). The measures were: 1) *COVID-19 related stress* – 27 items developed by the authors, e.g., loved one’s death from COVID, supply shortages; 2) *recent stressful life events* assessed by the List of Threatening Experiences [41]- 11 items, e.g., financial crisis, divorce; 3) *macro-stressors* – 11 items developed by the authors, e.g., police violence, healthcare access problems.

*Psychological Distress Symptoms* were measured at all study waves. For the present study, this construct was measured as a latent variable at W1 and W9 defined by past 7 day symptoms of depression and anxiety. Past 7 day symptoms of depression were measured by 7 items from the Center for Epidemiologic Studies – Depression scale (CESD) which correlate highly with the full CESD [42] (αs = 0.85 and 0.83). Past 7 day symptoms of anxiety were measured by 9 items from the Profile of Mood States [43] (αs = 0.88 and 0.88).

*Alcohol Misuse* was measured at all study waves. For the present study, this construct was measured as a latent variable at W1 and W9, defined by two indicators: frequency of past 12 month drinking to intoxication or consumption of 6 + drinks (0 = never to 7 = 5 or more times/week). These items have been used in other research to asses problematic alcohol use [44]. At W9, alcohol misuse variables were assessed for 2019, the year prior to W9, to avoid capturing increased alcohol misuse due to the COVID-19 pandemic which began in 2020.

#### Control variables

Control variables included gender (reference group: men), age, race/ethnicity (white or other/ > 1: reference group, Black, Asian/Pacific Islander, Hispanic), baseline occupational group (faculty: reference group, graduate student worker, administrative/clerical, and service/maintenance). Based on empirical evidence indicating WH exposure leads to increased financial stress for targets [45], we also controlled for W9 household income, using 11 categories ranging from 1 = “under $10,000” to 11 = “$250,000 or more.”

### Analysis plan

A series of hybrid path analyses were run in Mplus v.8.8 [46] to test the effects of prior exposure to WH, as indicated by latent class membership in each type of harassment, on psychological distress and alcohol misuse at follow-up. The MLR estimator was used to account for non-normal distributions of a subset of variables. Four models were fitted, with two separate models testing the effects of SH and GH on psychological distress, and two parallel models for alcohol misuse. In each model the latent dependent variable was regressed upon the W9 covariates (i.e., the stress index, income, and harassment), as well as a latent variable for that outcome in prior years, the harassment class, and the control variables. At the same time, the W9 covariates, the prior outcome variables, and harassment class were all regressed upon the exogenous demographic control variables. Bootstrapped indirect effects of harassment class were estimated as part of model fitting. Residual variances of the endogenous covariates were permitted to correlate. Gender and racial/ethnic differences were tested for all models but were not significant for all but one exception. We present models for the full sample, and for the exception we provide additional details about racial/ethnic differences (i.e., white versus all others).

Missing data analyses suggested that data were not missing completely at random (MCAR), Little’s MCAR χ^2^ (522) = 698.80, *p* < 0.001. Missingness on the W9 dependent variables was primarily attributable to attrition (i.e., missing data at one or more assessment between W2 and W8; Cramer’s V = 0.37). Additionally, those who were missing data at W9 were disproportionately likely to be male (Cramer’s V = 0.08) and had lower past year frequency of intoxication at W1 compared to W9 respondents (η^2^ = 0.002). There were no significant differences between W9 responders and non-responders for other study variables. Collectively, this suggested that data were missing at random (MAR), and when data are MAR, full information maximum likelihood (FIML) can generate unbiased estimates when the variables explaining missingness are modeled [47]. In this instance, we modeled only the demographic predictors of attrition and missingness in all models. We did not control for frequency of intoxication in the model for psychological distress symptoms, as its exceptionally small effect size indicates that the modest significant difference between those with and without W9 data was not meaningful. Similarly, we did not control for SH class in models considering GH, owing to the collinearity of these two class variables. Because we were able to employ FIML, n = 2352 cases were included in our models (N = 2492—72 known deceased – 68 missing information on 1 + demographic variables).

The primary indicator of acceptable model fit was a nonsignificant chi-square fit statistic (χ^2^). As chi-square statistics are sensitive to sampling fluctuation, comparative fit indices (CFIs) larger than 0.95, root mean square errors of approximation (RMSEAs) smaller than 0.06, or 90% confidence intervals (CIs) that contain 0.06, and standardized root mean square residuals (SRMRs) smaller than 0.08 were also used to indicate sufficient fit [48].

## Results

### Preliminary analyses

Descriptive statistics are provided for all study variables in Table 1. Approximately 1/3 of the sample were in the chronic class for each type of harassment. The SH and GH class variables were moderately and positively correlated (*r* = 0.45, *p* < 0.001). Membership in each chronic harassment class was associated with high levels of all indicators of psychological distress symptoms and alcohol misuse.

**Table 1:** Descriptive statistics for analytic sample, *n* = 2352

Variable	*n*	*M* (*SD*) / %	Range
Gender			
Women	1268	53.9%	
Men	1084	46.1%	
Race/ethnicity			
Black	503	21.4%	
White	1210	51.4%	
Hispanic	185	7.9%	
Asian/Pacific Islander	403	17.1%	
Other/ > 1	51	2.2%	
Occupational Group at W1			
Faculty	704	29.9%	
Clerical/administrative	521	22.2%	
Service/maintenance	267	11.4%	
Graduate student worker	860	36.6%	
Age W1	2352	39.89 (11.43)	20—86
Chronic Sexual Harassment Class	2329	33.1%	0—1
Chronic Generalized Harassment Class	2314	33.4%	0—1
Depression W1	2229	3.80 (4.13)	0—21
Anxiety W1	2244	7.65 (6.18)	0—36
Drinking to Intoxication W1	2321	.51 (.96)	0—7
6 + Drinks W1	2318	.56 (1.15)	0—7
Income W9	855	8.17 (2.39)	1—11
Stress Index W9	862	10.27 (4.73)	0—37
Depression Symptoms W9	873	3.26 (3.53)	0—21
Anxiety Symptoms W9	870	6.38 (5.39)	0—32
Drinking to Intoxication W9	888	.75 (1.42)	0—7
6 + Drinks W9	887	.51 (1.31)	0—7

*W9 =* Wave 9, *n* = 921. At W1, race and ethnicity were asked as a single question

### Psychological distress symptoms models

#### Sexual harassment

**Fig. 1:**
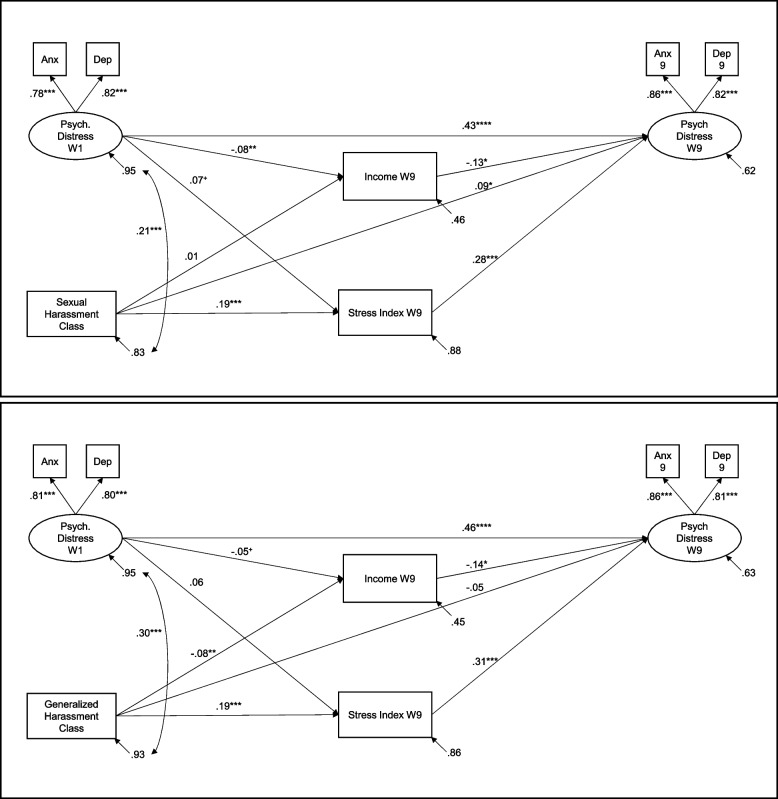
Effects of sexual and generalized harassment on recent psychological distress symptoms. Psych distress = psychological distress symptoms; Anx = Symptoms of anxiety; Dep = Symptoms of depression; W9 = Wave 9. All coefficients are standardized. + *p* < .10, * *p* < .05, ** *p* < .01, *** *p* < .001

Model fit was acceptable, X^2^ (23) = 67.12, *p* < 0.001, CFI = 0.979, RMSEA = 0.029, 90% CI [0.021, 0.037], SRMR = 0.053. Model results are presented in the top panel of Fig. 1, and coefficients for the control variables are reported in the top panel of Table 2. High prior psychological distress symptoms predicted low income and psychological distress symptoms at W9. High income predicted low psychological distress symptoms at W9. Membership in the chronic SH class was associated with high stress and high psychological distress symptoms at W9. In turn, high stress was associated with high psychological distress symptoms at W9. SH class had a significant indirect effect on psychological distress symptoms via high stress, β = 0.052, *p* < 0.001, 95% CI [0.027, 0.078].

**Table 2:** Coefficients for control variables

Control Variable	Prior Distress Symptoms	Har. Class	Income W9	Stress Index W9	Distress Symptoms W9
	Psychological Distress and Sexual Harassment				
Gender	-.04	-.01	.08**	-.05	-.05
Age	-.18***	-.40***	-.28***	-.18**	.07
Black	.03	-.04	-.16***	.15**	-.10*
Hispanic	-.02	-.05*	-.07*	.05	-.02
Asian	-.04	-.22***	.02	-.04	-.12*
Clerical/admin	-.08*	.05	-.53***	-.03	-.04
Student	.03	-.06	-.18***	.02	.08
Service/maintenance	-.03	.14***	-.45***	-.00	.01
	Psychological Distress and Generalized Harassment				
Gender	-.04	-.04	.07**	-.04	-.05
Age	-.18***	-.14***	-.29***	-.20***	.03
Black	.02	-.03	-.16***	.15**	-.11^+^
Hispanic	-.02	.00	-.07*	.05	-.03
Asian	-.04	-.09**	.01	-.06	-.14**
Clerical/admin	-.08**	.16***	-.51***	-.06	-.03
Student	.03	-.05	-.18***	.02	.07
Service/maintenance	-.03	.19***	-.43***	-.01	.03
	Alcohol Misuse and Sexual Harassment				
Gender	.14***	-.01	.08**	-.05	-.01
Age	-.27***	-.26***	-.28***	-.21***	-.02
Black	-.11***	-.04	-.16***	.15**	.03
Hispanic	-.08**	-.06*	-.07*	.06	.08
Asian	-.29***	-.28***	.01	-.08	-.06
Clerical/admin	.07*	.05	-.52***	-.04	.00
Student	.04	-.06	-.18***	.02	.06
Service/maintenance	.17***	.15***	-.44***	-.01	.11^+^
	Alcohol Misuse and Generalized Harassment				
Gender	.14***	-.04	.08**	-.05	-.01
Age	-.27***	-.16***	-.29***	-.23***	-.02
Black	-.11**	-.03	-.17***	.15**	.04
Hispanic	-.08***	.00	-.07*	.05	.08
Asian	-.29***	-.09**	.00	-.06	-.07
Clerical/admin	.07*	.16***	-.50***	-.07	-.00
Student	.04	-.05	-.18***	.02	.06
Service/maintenance	.17***	.19***	-.42***	-.02	.11^+^

All coefficients are standardized. Har. Class = Harassment Class. ^+^ *p* < .10, * *p* < .05, ** *p* < .01, *** *p* < .001. Reference categories: race/ethnicity = white or other/>1; W1 occupation = faculty; gender = male

#### Generalized harassment

Model fit was acceptable, X^2^ (23) = 69.82, *p* < 0.001, CFI = 0.978, RMSEA = 0.029, 90% CI [0.022, 0.037], SRMR = 0.042. Model results are presented in the bottom panel of Fig. 1, and coefficients for the control variables are reported in the second panel of Table 2. As before, high prior psychological distress symptoms predicted high psychological distress symptoms at W9 and high income predicted low psychological distress symptoms at W9. However, the association between prior distress symptoms and W9 income was not significantly different from zero. Membership in the chronic GH group was associated with low income and high stress, and in turn, high stress was associated with high psychological distress symptoms at W9. GH class had a significant indirect effect on psychological distress symptoms via high stress, β = 0.058, *p* < 0.001, 95% CI [0.025, 0.090].

### Alcohol use models

#### Sexual harassment

**Fig. 2:**
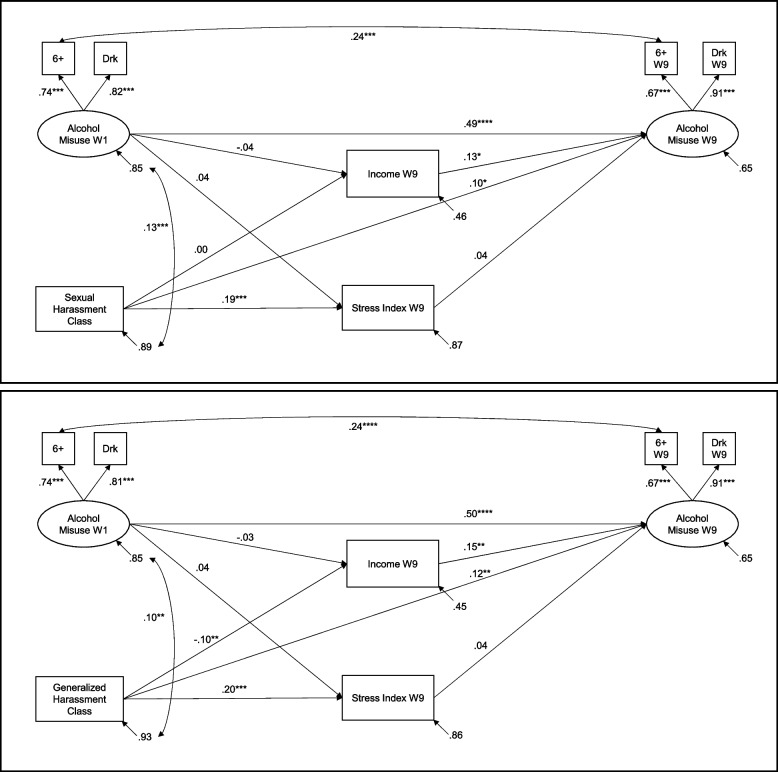
Effects of sexual and generalized harassment on recent alcohol misuse. 6 +  = Past 12 months frequency of six or more drinks on one occasion; Drk = Past 12 months number of times drunk/intoxicated; W9 = Wave 9. All coefficients are standardized. + *p* < .10, * *p* < .05, ** *p* < .01, *** *p* < .001

Initial model fit was not acceptable, X^2^ (23) = 216.45, *p* < 0.001, CFI = 0.923, RMSEA = 0.060, 90% CI [0.058, 0.07], SRMR = 0.081. Adding an autoregressive residual correlation between the indicators for 6 + drinks improved model fit, X^2^ (22) = 190.00, *p* < 0.001, CFI = 0.933, RMSEA = 0.057, 90% CI [0.050, 0.065], SRMR = 0.075. Model results are presented in the top panel of Fig. 2, and coefficients for the control variables are reported in the third panel of Table 2. High prior alcohol misuse predicted only alcohol misuse at W9. Membership in the chronic SH class was associated with high stress and with high levels of alcohol misuse in W9. High income was linked to high levels of alcohol misuse at W9. There were no significant indirect effects of SH class on heavy drinking via stress or income at W9.

#### Generalized harassment

As before, initial fit of the GH model for alcohol misuse was not acceptable, X^2^ (23) = 212.87, *p* < 0.001, CFI = 0.923, RMSEA = 0.059, 90% CI [0.052, 0.067], SRMR = 0.081. Model fit was improved through making the same adjustment as in the prior model, which improved model fit, X^2^ (22) = 187.92, *p* < 0.001, CFI = 0.933, RMSEA = 0.057, 90% CI [0.049, 0.064], SRMR = 0.075. Model results are presented in the bottom panel of Fig. 2, and coefficients for the control variables are reported in the fourth panel of Table 2. As before, high prior alcohol misuse predicted only alcohol misuse at W9. Membership in the chronic GH harassment group was associated with lower income levels, and with high levels of stress and alcohol misuse in W9. High income was linked to high levels of alcohol misuse at W9. Generalized harassment class had a trend-level indirect effect on heavy drinking via income, β = -0.015, *p* = 0.055, 95% CI [-0.027, 0.000].

Multigroup model comparisons indicated that a subset of this model’s coefficients varied by race/ethnicity, constrained model X^2^ (69) = 173.86, *p* < 0.001, estimated model X^2^ (36) = 122.39, *p* < 0.001, ΔX^2^ (33) = 51.47, *p* < 0.05. Follow-up analyses indicated that there were racial/ethnic differences for two main model paths. The path from GH to income was weaker for white respondents (β = -0.02, *p* = 0.87) than it was for non-white respondents (β = -0.17, *p* < 0.001), Δχ2 (1) = 12.78, *p* < 0.001. Additionally, the path from W1 alcohol misuse to the W9 stress index was stronger for white respondents (β = 0.12, *p* = 0.036) than it was for non-white respondents (β = -0.11, *p* = 0.062), Δχ2 (1) = 6.00, *p* < 0.025. Eight additional paths between demographic control variables and other model variables differed significantly across the two groups. As these are not the focus of the present investigation, we report these in Table S1 (see Supplementary Material).

We conducted a sensitivity analysis to assess whether including W9 SH or GH changed model results. We originally excluded W9 harassment from our alcohol models because our measures of WH referenced a time period *after* 2019 alcohol misuse would have occurred. However, this was not an issue for our psychological distress symptoms models, which measured past 7 day symptoms. We retested each model including W9 SH or GH. W9 harassment did not significantly impact model coefficients or results for either psychological distress symptoms or alcohol misuse (i.e., these variables did not predict either dependent variable, and its inclusion did not weaken or strengthen any other model paths). Given that it had no impact, we retained our models that exclude W9 harassment variables.

## Discussion

With this study, we sought to examine the long-term impact of prior exposure to WH on recent health-related outcomes in a US sample. Our findings extend existing prospective investigations of WH by documenting the effects of SH and GH over a career-spanning approximately 25 year time period. Specifically, our results show that chronic exposure to SH and GH have significant long-term effects on recent psychological distress and alcohol misuse, accounting for baseline levels of these variables. The long-term impact of WH on mental and behavioral health is important because of its implications for workplace health and safety as well as future population health, given that approximately 1/3 of our sample experienced chronic SH or GH. Additional research is needed to determine if chronic harassment is as prevalent in representative samples.

Interestingly, the strongest effects of prior WH exposure on recent psychological distress symptoms were through increased levels of other current stressors. This is consistent with Pearlin’s stress proliferation framework, whereby serious stressors are thought to bring about additional acute and chronic stressors which further contribute to health inequalities [49]. Thus, WH may impact mental and physical health long-term through initiating a cascade of other life stressors (e.g., job loss, loss of income) that create additional strain by incrementally diminishing available resources for dealing with stress. Our findings that those in the chronic GH class had significantly lower household income at W9 also lend support to this notion. Persistence of the effects of harassment over the 13–14 year gap between the prior study’s last harassment measurement point and our follow-up at W9 is compelling. Only about 56% of our W9 respondents had worked for pay in the past 12 months, suggesting that exposure to WH can continue to adversely impact psychological and behavioral health even if one is no longer in the workforce and no longer exposed to WH.

The results show that prior chronic WH exposure is directly associated with current heavier drinking, and that these effects are stronger than the effects of current stressors. Although the direct effects of prior WH exposure on psychological health and alcohol misuse were relatively small, they are still important because the literature has shown that even moderate use of alcohol is associated with premature mortality from diseases (i.e., cancer) and increased burden of disease from health conditions made worse by alcohol use [50]. Furthermore, workplace harassment is a more preventable stressor than other life stressors (e.g., death of a loved one), as it can be directly addressed through organizational policy and law. This study is also important because it is the first study of the effects of WH on alcohol misuse to follow respondents into later adulthood, a time when alcohol abuse in particular increases risk for mortality [51]. In addition to taxing the immune system [52], heavier drinking over time can also manifest as a chronic stressor by straining friendships and marriages [53] or leading to poor job performance, lowered productivity and potential job loss [54]. Further research is needed to determine whether effects of WH and other work-related psychosocial hazards on drinking behavior and associated proliferation of other life stressors long-term ultimately increase risk for mortality.

### Strengths and limitations

This study capitalized on a unique opportunity for long-term follow-up of a diverse sample representing a wide variety of job types at baseline. Although the study sample is not a representative sample of the entire U.S. population, we were still able to demonstrate that the negative effects of WH hold across occupation types for both men and women and for people of different racial/ethnic identities. We were also able to demonstrate persistence of the impact of harassment on outcomes over time. In addition to our longitudinal design, other strengths include consideration of other stressors that might impact outcomes, and FIML estimation of effects which allowed us to use all available data and maximized power for our models.

As previously mentioned, our sample was initially drawn from a single workplace in 1996 and is not be representative of U.S. workers in general, so findings may not be generalizable. Our data were also solely self-report, which increases risk for common method bias. There was also systematic attrition after W1. As this was primarily attributable to demographic factors, we were able to minimize potential bias through retaining all cases, and by controlling for these variables while employing FIML estimation methods. Future research in this area should apply population-based random sampling of employed individuals and incorporate psychiatric interviews for assessment of presence or absence of major depressive disorder, anxiety disorder, and alcohol or substance use disorders. This will allow for a more accurate evaluation of the long-term impacts of exposure to workplace harassment for U.S. workers. However, such research would require a commitment by U.S. funding bodies to invest in large-scale prospective epidemiologic research, which is currently beyond the scope of NIH fundable research.

## Conclusions

This is the first study to examine the effects of WH over an approximately 25 year period. Given our findings that exposure to WH can have long-term effects on worker health, stronger enforcement of SH law and enactment of laws to prevent GH/workplace bullying are crucial for the protection of worker health. Additionally, employers should enforce existing sexual harassment policies and institute policies that explicitly prohibit generalized harassment or bullying in the workplace. Policies should include clear reporting procedures and clear penalties for policy violations. More longitudinal research is also warranted to test long-term health effects of other workplace psychosocial hazards for workers.

## Supplementary Information

**Additional file 1: Table S1. **Moderated Paths in the Generalized Harassment-Alcohol Model.

## Data Availability

The datasets used and/or analysed during the current study are available from the corresponding author on reasonable request.

## Abbreviations

GH: Generalized workplace harassment/bullying
SH: Sexual harassment
FIML: Full information maximum likelihood
U.S.: United States
WH: Workplace Harassment
W1: Wave 1 (baseline)
W2: Wave 2
W7: Wave 7
W8: Wave 8
W9: Wave 9
