# Supplementary material for: Effects of chronic workplace harassment on mental health and alcohol misuse: a long-term follow-up
Source: BMC Public Health. 2023 Jul 26;23:1430. doi: 10.1186/s12889-023-16219-0 (PMC10373226; doi:10.1186/s12889-023-16219-0)
Supplement: Supplementary file 1 — Additional file 1: Table S1. Moderated Paths in the Generalized Harassment-Alcohol Model. [file 12889_2023_16219_MOESM1_ESM.pdf]

Table S1

Moderated Paths in the Generalized Harassment-Alcohol Model

Path		Coefficient		Comparison
Predictor	Outcome	White	Other	$\Delta\chi^2$ (1)
Gender	Alcohol Misuse W9	-.06	.13	4.73*
Age	Stress Index	-.33***	-.09	6.00*
Student	Income	-.31***	-.01	9.37**
Gender	NSH Class	-.09**	.01	3.89*
Student	NSH Class	.01	-.18**	6.19*
Service/maintenance	NSH Class	.20***	.15**	4.49*
Age	Alcohol Misuse W1	-.30***	-.21***	4.45*
Service/maintenance	Alcohol Misuse W1	.12*	.27***	4.57*

Note. * $p < .05$, ** $p < .01$, *** $p < .001$.
